# Capacity-building for a strong public health nutrition workforce in low-resource countries

**DOI:** 10.2471/BLT.16.174912

**Published:** 2017-04-05

**Authors:** Hélène Delisle, Roger Shrimpton, Sonia Blaney, Lisanne Du Plessis, Stephen Atwood, David Sanders, Barrie Margetts

**Affiliations:** a Department of Nutrition, University of Montreal, PO Box 6128 Downtown Station, Montreal, Quebec, H3C 3J7, Canada.; b Department of Global Community Health and Behavioral Sciences, Tulane School of Public Health and Tropical Medicine, New Orleans, United States of America.; c École des Sciences des Aliments, de Nutrition et d'Études Familiales, Université de Moncton, Moncton, Canada.; d Faculty of Medicine and Health Sciences, Stellenbosch University, Stellenbosch, South Africa.; e School of Global Studies, Thammasat University, Bangkok, Thailand.; f School of Public Health, University of the Western Cape, Cape Town, South Africa.; g Faculty of Medicine, University of Southampton, Southampton, England.

Neglected for several decades, nutrition is now firmly on the development agenda. Important landmarks are the initiation of the Scaling Up Nutrition movement in 2010; the adoption by the World Health Assembly of the Comprehensive Implementation Plan for Maternal, Infant and Young Child Nutrition in 2014; and the World Health Organization’s (WHO) Global Action Plan for the Prevention and Control of Noncommunicable Diseases for 2013–2020. Public health nutrition has to meet multiple new challenges, including the shift from the millennium development goals to the sustainable development goals (SDGs), together with growing issues such as climate change, globalization, urbanization, socioeconomic disparities, migration and wars.^1^ The nutrition problems of low-resource countries are also becoming more complex due to the double burden of undernutrition and overnutrition. Obesity and other nutrition-related chronic diseases have become a health priority in most of the world, including low- and middle-income countries.^2^ Nutrition is at the core of prevention, and even management, of chronic diseases. Meanwhile, in sub-Saharan Africa and parts of Asia, maternal and child undernutrition (including micronutrient deficiencies) remains the first cause of mortality and morbidity.^3^ To further boost efforts towards the elimination of all forms of malnutrition, the United Nations declared 2016–2026 the Decade of Action on Nutrition.^4^

Despite some progress, efforts to alleviate malnutrition, whether under- or overnutrition, are hampered by countries’ lack of capacity in public health nutrition, including an insufficient and poorly qualified workforce. This paper highlights current issues and challenges in public health nutrition in low- and middle-income countries and shares recommendations for the development of this workforce. It is primarily based on the work of the World Public Health Nutrition Association capacity-building taskforce, while also drawing on relevant data from published material. Background documents are available on the Association’s website (http://wphna.org), and a full report has been published elsewhere.^5^

The need for capacity-building in public health nutrition at the individual, organizational and systemic levels is reflected in the number of working groups and studies on this issue, particularly for Africa.^6^^–^^12^ The Scaling Up Nutrition initiatives, aimed at achieving national coverage of essential nutrition interventions, are constrained by the lack of human resources in nutrition in several countries.^13^ The Comprehensive Implementation Plan resolution explicitly calls for implementing a comprehensive approach to capacity-building, including workforce development.^14^ WHO has stressed the need to train not only physicians and nurses but also nutritionists (and other health workers) to achieve the Comprehensive Implementation Plan goals. Unfortunately, the Global Strategy for Health Workforce Development does not include nutrition professionals.^15^

Malnutrition is rooted in poverty and food insecurity.^16^^,^^17^ Nevertheless, even when these issues are addressed through money and food transfers, adding a nutrition education component targeting the poorest population can increase the positive impact of an intervention on child chronic malnutrition.^18^ Such efforts, however, are hampered by a shortage in numbers, skills and geographical coverage of nutrition workers in low- and middle-income countries.^6^ The recommended density of nutritionists per 5 million population, based on the Manila report,^19^ is 100–500 at bachelor degree or licence level qualifications, 10–50 at masters level and 5–25 at doctorate level. For West Africa alone, the estimated need is 700 nutrition graduates per 5 million population, while the current output is about 250, with a more pronounced gap for nutritionists qualified at bachelor than masters level, and in French-speaking countries compared with English-speaking ones.^8^ In high-income countries, current ratios are much higher, ranging from 1250 to 2800 registered nutritionists or dietitians per 5 million population in Canada and the United States of America.^20^^,^^21^

Several factors may contribute to this scarcity of nutrition professionals in low- and middle-income countries. One reason is a lack of understanding of the role of public health nutrition in the prevention and management of the various forms of malnutrition. Another is that low-income countries tend to prioritize doctors and nurses (and sometimes also frontline workers) within their meagre health workforce expenditures. The complexity of nutrition as a discipline and practice tends to be overlooked; doctors, nurses and community health workers need specific preparation^9^ or guidance to deliver the nutrition services that health facilities are expected to deliver (as in Indonesia^22^). When nutrition professionals are unavailable for field programmes, international nongovernmental organizations (NGOs) may hire other workers, whose nutrition competencies may be highly variable.

Another issue is that in lower-income countries undernutrition is a higher priority for interventions than nutrition-related chronic diseases. The latter are currently escalating in these countries. Both food system changes, at the level of production, processing and distribution, and behaviour change communication are needed to reorient the nutrition transition, and nutritionists have a major role to play in this regard. Studies on nutrition workforce capacity conducted in West Africa confirm this, and also highlighted the severe shortage of trained nutrition professionals (except in Nigeria and Ghana).^23^ Other weaknesses were that training emphasized food science and the treatment of severe undernutrition at the expense of public health nutrition; teaching was predominantly theoretical; and nutrition training of other health professionals was quite poor. In addition, because of a shortage of nutritionists, the bulk of the nutrition interventions were done by health workers (e.g. nurses or community health workers) who lacked the skills to provide quality nutrition services. The fact that nutritionists often had only a few weeks or months of training drew attention to the lack of regulation in the nutrition profession in the region.^23^ In Asia, a study in three countries showed that the nutrition knowledge of health professionals was outdated and that nutrition competencies were limited to curative activities (e.g. correcting nutritional deficiencies or treating severely malnourished children). The limited capacity of trainers was also noted.^24^ In the era of the SDGs, nutrition professionals need to be trained to apply a systems thinking approach, with nutrition being linked to broader systems of health, food and the environment.^6^

How can we build this public health nutrition workforce? Several initiatives have been taken to assess and strengthen the capacity of nutrition workforces. However, substantive action is still needed with the support of WHO and other actors, as shown in the recommendations (Box 1). The International Union of Nutrition Sciences and the International Malnutrition Taskforce are addressing capacity development in nutrition. The eNutrition Academy was created in 2014 by a consortium of international nutrition organizations to offer free e-learning modules. The United Nations Children’s Fund and partners have developed e-learning modules on nutrition programming for Africa, and launched an initiative to assess and improve nutrition capacity in West Africa in 2010. The World Public Health Nutrition Association capacity-building taskforce, formed in 2008, held several workshops and developed various tools, including capacity assessment and competency frameworks to be used for curriculum development.

Box 1 **Recommendations for strengthening the nutrition workforce in low- and middle-income countries**

- More nutritionists need to be trained at the undergraduate level to carry out the bulk of nutrition intervention work.
- A core of specialized nutritionists qualified in public health nutrition at masters level needs to be formed in every country to work at national and district levels.
- More and up-to-date public health nutrition has to be integrated into the curriculum of medical and nursing studies, as well as in the training programmes of nutritionists.
- In-service training in nutrition and in the management of nutrition programmes is required for scaling-up nutrition efforts. This calls for initial in-service training of available staff, supervisors and trainers.
- Hybrid training programmes, which combine distance training and periodic in-person sessions with tutors and peers, are a cost-effective approach for pre-service and in-service training as well as for continuous education of nutrition professionals.
- Competency standards for nutrition job descriptions, and curricula and accreditation schemes, should be developed and harmonized at the regional level, with international support.
- Development of a national nutrition workforce should be an integral component of national nutrition and workforce development plans, with adequate funding secured by governments and other relevant actors including nongovernmental organizations.

**Fig. 1:**
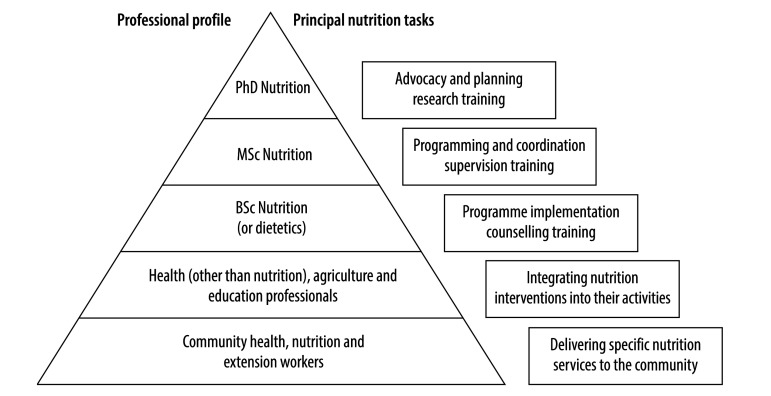
The nutrition workforce pyramid

BSc: bachelor-level degree; MSc masters-level degree; PhD: doctorate-level degree.

Source: adapted from Shrimpton et al.^7^

The nutrition workforce is best portrayed as a pyramid, representing the numbers, level of training and occupational profiles at various levels (Fig. 1). The base consists of community health, nutrition and extension workers, who need vocational or on-the-job training to deliver some nutrition services directly to populations (e.g. child growth monitoring and promotion). The upper levels are nutritionists (and dietitians where relevant) with different levels of university training for different roles: from implementation of programmes and nutrition counselling at individual and community level, through programming and coordination, up to planning, advocacy and research at national level. Bachelor-level nutritionists and dietitians could perform most of the required nutrition activities at country and district level, and their competence could be maintained through continuous education, as is the case in several high-income countries. Having more masters-level than bachelor-level nutritionists, as is currently the case in several African countries,^11^ for instance, is not cost-effective. At all levels, nutrition professionals can play an important role in training in a cascade fashion.

Although it requires sustained efforts, training can be regarded as the easy part of nutrition workforce development in low- and middle-income countries. The core technical and horizontal skills that public health nutritionists (masters level) need to acquire in the areas of intervention management, capacity-building and research have been proposed, along with assessment indicators.^25^ More challenging steps are recognition of the nutrition profession and its regulation, opening up government jobs for nutrition graduates and financing local training programmes and nutritionists’ salaries in the public sector. A current problem is that short, but unsustainable, training programmes are often offered by external agencies to fit the needs of specific development projects. These function independently from local universities, which remain resource-poor. Nutrition graduates may end up unemployed unless hired by international groups. While public financing is the most sustainable source of funding for higher education, other actors ‒ particularly the large NGOs that hire nutritionists for their programmes ‒ should contribute, for instance, by supporting training programmes or offering scholarships. Quality education is expensive and the funding issue is real,^26^ even if modern technology allows costs to be reduced through online training. Arguably, distance education is not sufficient. The language divide is another barrier to capacity-strengthening, although this is not an issue unique to nutrition. However, the concern is that it hinders training, action and research for improved nutrition. This may be one of the reasons why French-speaking African countries, for example, lag behind English-speaking Africa in nutrition workforce capacity.^23^

The underlying causes of malnutrition, and hence sustained solutions to the problem, lie to a large extent in the non-health sectors. Nutrition therefore has to be addressed not only by other health professionals, but also by agriculture and education professionals and field workers, who need to integrate relevant nutrition tasks into their professional activities (such as orienting food production towards meeting the population’s nutrition requirements or teaching healthy eating to schoolchildren; Fig. 1). Strong workforces in nutrition are essential not only for scaling up nutrition programmes but also for implementing nutrition-sensitive interventions in these sectors. Nutrition professionals are needed in sufficient numbers to ensure adequate and sustainable training and monitoring of those who deliver nutrition-specific or nutrition-sensitive services to communities.
